# Supplementary material for: Mesoscale Simulations of Polymer Solution Self-Assembly: Selection of Model Parameters within an Implicit Solvent Approximation
Source: Polymers (Basel). 2021 Mar 19;13(6):953. doi: 10.3390/polym13060953 (PMC8003785; doi:10.3390/polym13060953)
Supplement: Supplementary file 1 [file polymers-13-00953-s001.pdf]

Supplementary Materials

Mesoscale Simulations of Polymer Solution Self-Assembly: Selection of Model Parameters within an Implicit Solvent Approximation

Juhae Park[1,2], Abelardo Ramírez-Hernández[3,4,*], Vikram Thapar[1,2,*], Su-Mi Hur[1,2,*]

[1] Department of Polymer Engineering, Graduate School, Chonnam National University, Gwangju 61186, South Korea

[2] Alan G. MacDiarmid Energy Research Institute & School of Polymer Science and Engineering, Chonnam National University, Gwangju 61186, South Korea

[3] Department of Biomedical Engineering and Chemical Engineering, The University of Texas at San Antonio, San Antonio, TX 78249, USA

[4] Department of Physics and Astronomy, The University of Texas at San Antonio, San Antonio, Texas 78249, USA

*E-mail : shur@chonnam.ac.kr (S.-M.H.); thapar.09@gmail.com (V.T.); abelardo.ramirez-hernandez@utsa.edu (A.R.-H.)

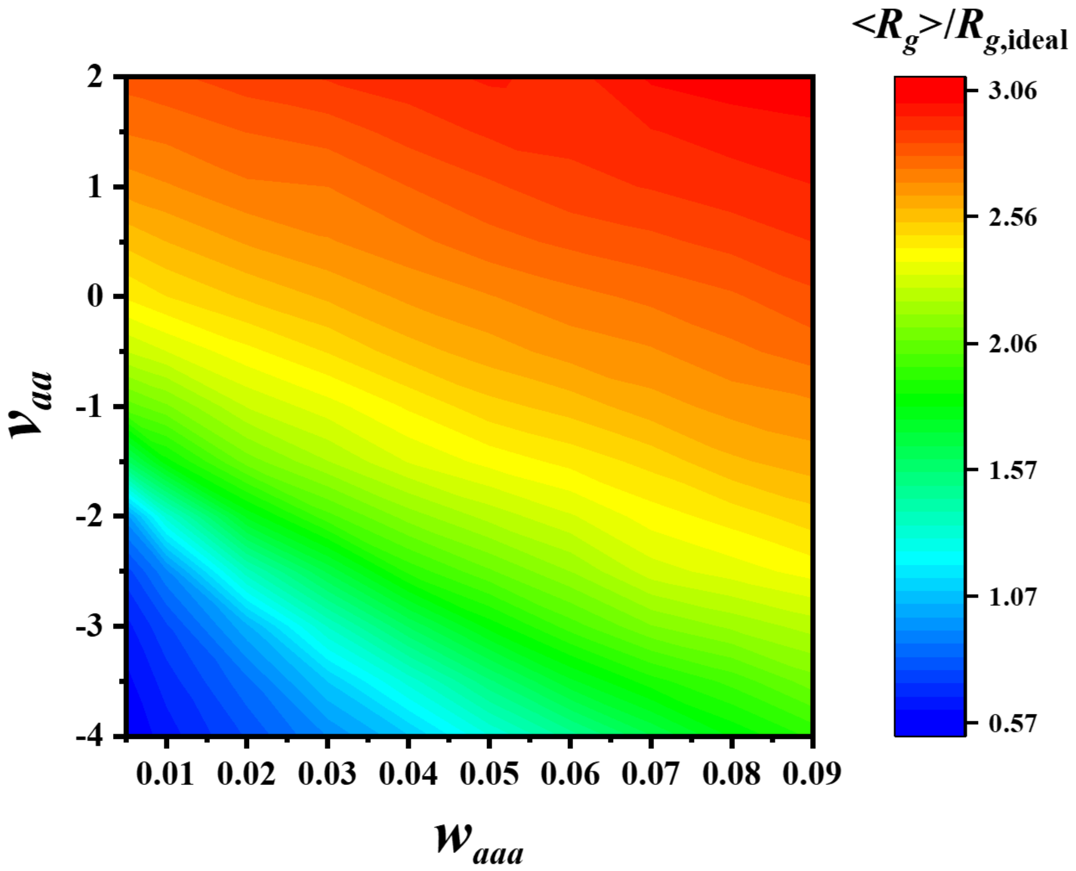

Figure S1. Single chain size variation upon model parameters, virial coefficients.

Colormap of ratio of average radius of gyration at various (v_{aa},w_{aaa}) to the size of an ideal chain, $<R_g>/R_{g,ideal}$ for a chain length of $N = 64$, obtained from MC-GL single-chain simulations for given sets of virial coefficients(v_{aa}, w_{aaa}).

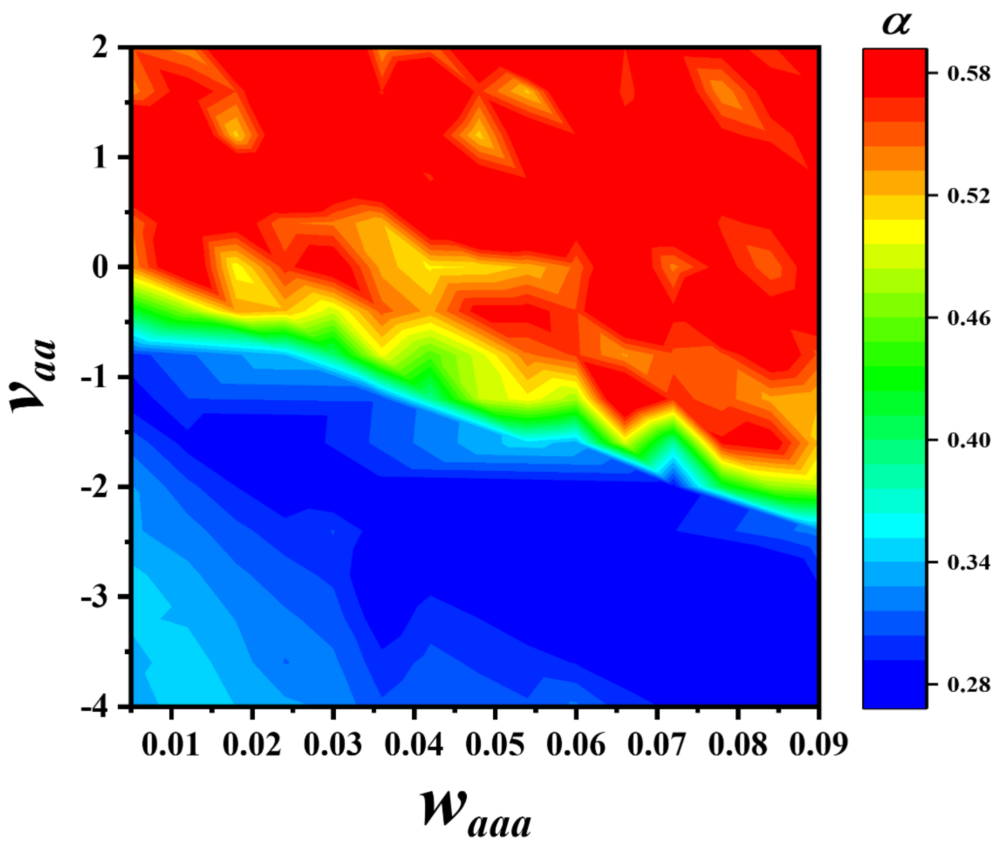

Figure S2. Relation of model parameters with solvent quality obtained from a single chain scaling analysis using MC-G simulations. Colormap of scaling exponent α in a power-law relation $\boldsymbol{R_g} \propto \boldsymbol{N^{\alpha}}$, which are extracted from single-chain MC-G simulations over various chain length N for given sets of virial coefficients (v_{aa}, w_{aaa}). The same procedures as MC-GL simulations are used for initial configuration preparation, simulation box size, MC cycles for equilibration, MC cycles over which R_g is averaged, values of virial coefficients (v_{aa}, w_{aaa}), etc. The value of grid discretization length, ΔL_G is set to be the same as ΔL_{GL} which is $0.16R_e$. Obtained colormap is very similar to Figure 1a obtained with MC-GL simulations with slight discrepancies in the locations of the poor, theta and good solvent regions.

Simulation details for interfacial tension calculation.

The interfacial tension (γ) of binary homopolymer blends was obtained by performing MD-GL simulations of a system containing two homopolymers, A and B. The initialization of the system was performed by placing 2880 A chains in the top half of the simulation box (along the z-direction) and 2880 B chains in the bottom half the simulation box. Each of the A and B chains has N (=64) coarse grained beads and is initialized with randomly positioned bonds between adjacent beads of bond length, b. The values of b and R_e are set as value of bond length and mean squared end to end distance of an ideal chain of 64 beads respectively. The box dimensions of $L_x = 3R_e$, $L_y = 3R_e$ and $L_z = 5R_e$ are used with periodic boundary conditions. The virial coefficients (v_{aa}, w_{aaa}) and (v_{bb}, w_{bbb}) are set to be same as (-1.6094,0.009338) and immiscibility is modeled in the cross second order virial coefficient[1-3]. The cross second order virial coefficient, v_{ab} contains the segregation strength between A and B, χN (Flory-Huggins parameter multiplied with degree of polymerization) and is defined as

$$v_{ab} = \frac{\chi N}{\rho'} + \frac{v_{aa} + v_{bb}}{2}$$

where ρ' here is equivalent to the equilibrium chain density at melt conditions, which is in this case is the summation of number of A and B chains divided by the volume of simulation box. The cross third-order virial coefficients are expressed as a weighted arithmetic mean of w_{aaa} and w_{bbb} and are explicitly written as

$$w_{aab} = \frac{2w_{aaa} + w_{bbb}}{3}, \qquad w_{bba} = \frac{w_{aaa} + 2w_{bbb}}{3}$$

The value of χN is varied from 5 to 60 and for each value, interfacial tension is estimated using the same procedures of initialization, equilibration and evaluation of tension via measuring pressure tensor as the one for surface tension.

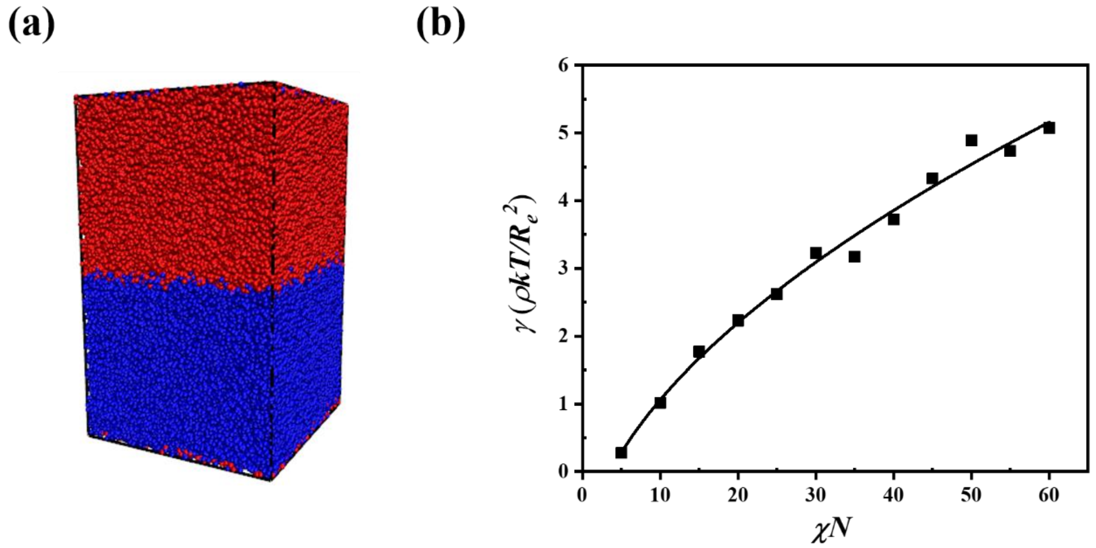

Figure S3. Macro-phase separated binary homopolymer blend. (a) Snapshot of the equilibriated configurations from MD-GL simulation of A/B homopolymer blend system of χN = 20. Red and blue represent A and B homopolymers of $N=64$. (b) Evaluated interfacial tension (γ) of the immiscible homopolymer blend as a function of χN varied from 5 to 60. Solid curve is power law fitting $y = A + Bx^C$ where A, B, and C is -1.42356, 0.71054, and 0.5437, respectively.

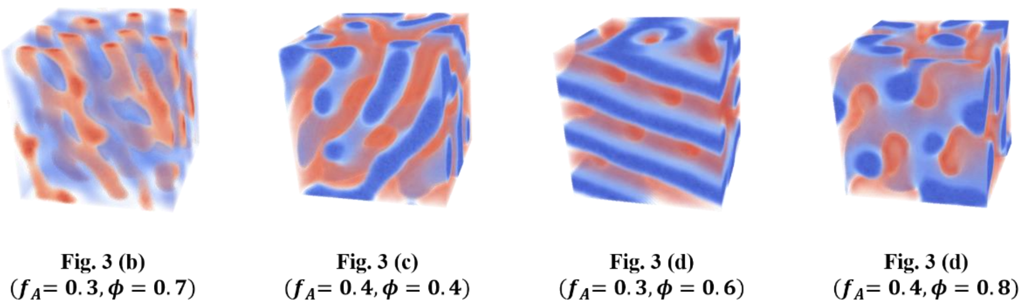

Figure S4. Snapshots of representative morphologies classified as bicontinuous phase in the phase diagram of AB BCPs solutions. A few representation snapshots of labeled bicontinuous phases in Figure 3(a)-(d) of the main text. The text underneath represents the location of a given snapshot in Figure 3. It includes the value of f_A, ϕ and subfigure number.

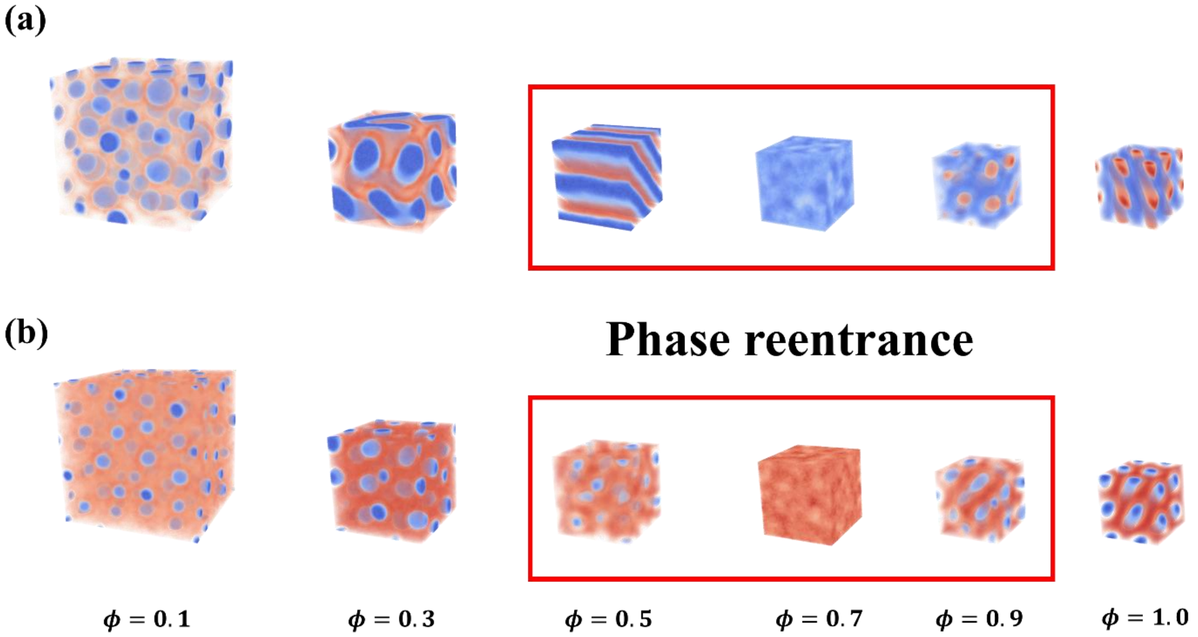

Figure S5. Simulation snapshots showing phase reentrance with polymer concentration ϕ change in block copolymer solution self-assembly. Observed morphologies at different ϕ values in the vertical lines of for (a) $f_A = 0.3$ and (b) $f_A = 0.7$ in Figure 3(c) of the main text. Figure 3(c) is the phase diagram of AB BCPs in the marginally poor solvent to the B block, while the A block is solvable. The phase reentrance locations depicting the transition from an ordered phase to a disordered one and back to an ordered state are indicated with red boxes for both subfigures.

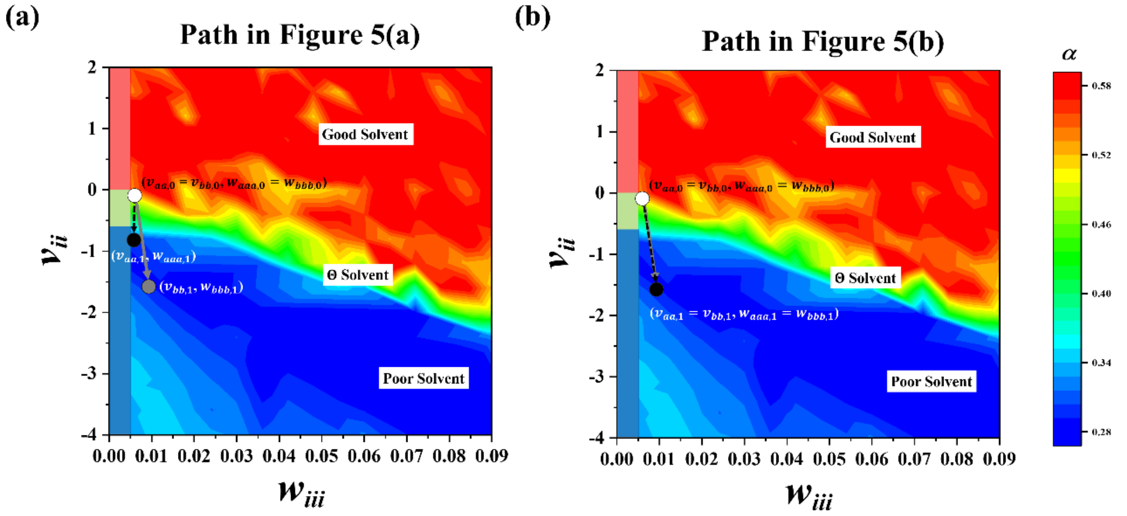

Figure S6. Trajectories of model parameters in emulsified BCP droplet simulations. For each of the subfigures in Figure 5, a corresponding subfigure of (v,w) parametric colormap of scaling exponent with marked virial coefficients, $(v_{aa,0}, w_{aaa,0})$, $(v_{bb,0}, w_{bbb,0})$, $(v_{aa,1}, w_{aaa,1})$ and $(v_{bb,1}, w_{bbb,1})$. A line connecting $(v_{aa,0}, w_{aaa,0})$ to $(v_{aa,1}, w_{aaa,1})$ and a line connecting $(v_{bb,0}, w_{bbb,0})$ to $(v_{bb,1}, w_{bbb,1})$ are shown in black dashed line and gray solid line respectively.

Reference

1. Hömberg, M.; Müller, M. Main phase transition in lipid bilayers: phase coexistence and line tension in a soft, solvent-free, coarse-grained model. *The Journal of chemical physics* **2010**, *132*, 04B609.
2. Wang, J.; Mu¨ller, M. Microphase separation of diblock copolymer brushes in selective solvents: Single-chain-in-mean-field simulations and integral geometry analysis. *Macromolecules* **2009**, *42*, 2251-2264.
3. Hur, S.-M.; Khaira, G.S.; Ramírez-Hernández, A.; Mu¨ller, M.; Nealey, P.F.; de Pablo, J.J. Simulation of defect reduction in block copolymer thin films by solvent annealing. *ACS Macro Letters* **2015**, *4*, 11-15.
